# Supplementary material for: A fault-intrusion-tolerant system and deadline-aware algorithm for scheduling scientific workflow in the cloud
Source: PeerJ Comput Sci. 2021 Nov 2;7:e747. doi: 10.7717/peerj-cs.747 (PMC8576554; doi:10.7717/peerj-cs.747)
Supplement: Supplemental Information 6 [file peerj-cs-07-747-s006.zip › Myproject1.1/docs/org/cloudbus/cloudsim/class-use/VmAllocationPolicy.html]

Uses of Class org.cloudbus.cloudsim.VmAllocationPolicy


JavaScript is disabled on your browser.


- Overview
- Package
- Class
- Use
- Tree
- Deprecated
- Index
- Help

- Prev
- Next

- Frames
- No Frames

- All Classes

## Uses of Class org.cloudbus.cloudsim.VmAllocationPolicy

- Packages that use VmAllocationPolicy

  | Package | Description |
  | --- | --- |
  |  |  |
  | --- | --- |
  | org.cloudbus.cloudsim |  |
  | org.cloudbus.cloudsim.examples.power |  |
  | org.cloudbus.cloudsim.network.datacenter |  |
  | org.cloudbus.cloudsim.power |  |
  | org.workflowsim |  |
- - ### Uses of VmAllocationPolicy in org.cloudbus.cloudsim

    Subclasses of VmAllocationPolicy in org.cloudbus.cloudsim

    | Modifier and Type | Class and Description |
    | --- | --- |
    |  |  |
    | --- | --- |
    | `class` | `VmAllocationPolicySimple` VmAllocationPolicySimple is an VmAllocationPolicy that chooses, as the host for a VM, the host with less PEs in use. |

    Methods in org.cloudbus.cloudsim that return VmAllocationPolicy

    | Modifier and Type | Method and Description |
    | --- | --- |
    |  |  |
    | --- | --- |
    | `VmAllocationPolicy` | Datacenter.`getVmAllocationPolicy()` Gets the vm allocation policy. |

    Methods in org.cloudbus.cloudsim with parameters of type VmAllocationPolicy

    | Modifier and Type | Method and Description |
    | --- | --- |
    |  |  |
    | --- | --- |
    | `protected void` | Datacenter.`setVmAllocationPolicy(VmAllocationPolicy vmAllocationPolicy)` Sets the vm allocation policy. |

    Constructors in org.cloudbus.cloudsim with parameters of type VmAllocationPolicy

    Constructor and Description

    `Datacenter(java.lang.String name,
    DatacenterCharacteristics characteristics,
    VmAllocationPolicy vmAllocationPolicy,
    java.util.List<Storage> storageList,
    double schedulingInterval)`

    Allocates a new PowerDatacenter object.
  - ### Uses of VmAllocationPolicy in org.cloudbus.cloudsim.examples.power

    Methods in org.cloudbus.cloudsim.examples.power that return VmAllocationPolicy

    | Modifier and Type | Method and Description |
    | --- | --- |
    |  |  |
    | --- | --- |
    | `protected VmAllocationPolicy` | RunnerAbstract.`getVmAllocationPolicy(java.lang.String vmAllocationPolicyName, java.lang.String vmSelectionPolicyName, java.lang.String parameterName)` Gets the vm allocation policy. |

    Methods in org.cloudbus.cloudsim.examples.power with parameters of type VmAllocationPolicy

    | Modifier and Type | Method and Description |
    | --- | --- |
    |  |  |
    | --- | --- |
    | `static Datacenter` | Helper.`createDatacenter(java.lang.String name, java.lang.Class<? extends Datacenter> datacenterClass, java.util.List<PowerHost> hostList, VmAllocationPolicy vmAllocationPolicy)` Creates the datacenter. |
    | `protected void` | RunnerAbstract.`start(java.lang.String experimentName, java.lang.String outputFolder, VmAllocationPolicy vmAllocationPolicy)` Starts the simulation. |
  - ### Uses of VmAllocationPolicy in org.cloudbus.cloudsim.network.datacenter

    Subclasses of VmAllocationPolicy in org.cloudbus.cloudsim.network.datacenter

    | Modifier and Type | Class and Description |
    | --- | --- |
    |  |  |
    | --- | --- |
    | `class` | `NetworkVmAllocationPolicy` NetworkVmAllocationPolicy is an VmAllocationPolicy that chooses, as the host for a VM, the host with less PEs in use. |

    Constructors in org.cloudbus.cloudsim.network.datacenter with parameters of type VmAllocationPolicy

    Constructor and Description

    `NetworkDatacenter(java.lang.String name,
    DatacenterCharacteristics characteristics,
    VmAllocationPolicy vmAllocationPolicy,
    java.util.List<Storage> storageList,
    double schedulingInterval)`

    Allocates a new NetworkDatacenter object.
  - ### Uses of VmAllocationPolicy in org.cloudbus.cloudsim.power

    Subclasses of VmAllocationPolicy in org.cloudbus.cloudsim.power

    | Modifier and Type | Class and Description |
    | --- | --- |
    |  |  |
    | --- | --- |
    | `class` | `PowerVmAllocationPolicyAbstract` The class of an abstract power-aware VM allocation policy. |
    | `class` | `PowerVmAllocationPolicyMigrationAbstract` The class of an abstract power-aware VM allocation policy that dynamically optimizes the VM allocation using migration. |
    | `class` | `PowerVmAllocationPolicyMigrationInterQuartileRange` The Inter Quartile Range (IQR) VM allocation policy. |
    | `class` | `PowerVmAllocationPolicyMigrationLocalRegression` The Local Regression (LR) VM allocation policy. |
    | `class` | `PowerVmAllocationPolicyMigrationLocalRegressionRobust` The Local Regression Robust (LRR) VM allocation policy. |
    | `class` | `PowerVmAllocationPolicyMigrationMedianAbsoluteDeviation` The Median Absolute Deviation (MAD) VM allocation policy. |
    | `class` | `PowerVmAllocationPolicyMigrationStaticThreshold` The Static Threshold (THR) VM allocation policy. |
    | `class` | `PowerVmAllocationPolicySimple` This a simple class representing a simple VM allocation policy that does not perform any optimization of the VM allocation. |

    Constructors in org.cloudbus.cloudsim.power with parameters of type VmAllocationPolicy

    Constructor and Description

    `PowerDatacenter(java.lang.String name,
    DatacenterCharacteristics characteristics,
    VmAllocationPolicy vmAllocationPolicy,
    java.util.List<Storage> storageList,
    double schedulingInterval)`

    Instantiates a new datacenter.

    `PowerDatacenterNonPowerAware(java.lang.String name,
    DatacenterCharacteristics characteristics,
    VmAllocationPolicy vmAllocationPolicy,
    java.util.List<Storage> storageList,
    double schedulingInterval)`

    Instantiates a new datacenter.
  - ### Uses of VmAllocationPolicy in org.workflowsim

    Constructors in org.workflowsim with parameters of type VmAllocationPolicy

    Constructor and Description

    `DatacenterExtended(java.lang.String name,
    DatacenterCharacteristics characteristics,
    VmAllocationPolicy vmAllocationPolicy,
    java.util.List<Storage> storageList,
    double schedulingInterval)`

- Overview
- Package
- Class
- Use
- Tree
- Deprecated
- Index
- Help

- Prev
- Next

- Frames
- No Frames

- All Classes
